# ROS Scavenging Effect of Selected Isoflavones in Provoked Oxidative Stress Conditions in Human Skin Fibroblasts and Keratinocytes

**DOI:** 10.3390/molecules29050955

**Published:** 2024-02-22

**Authors:** Magdalena Wójciak, Piotr Drozdowski, Aleksandra Ziemlewska, Martyna Zagórska-Dziok, Zofia Nizioł-Łukaszewska, Tomasz Kubrak, Ireneusz Sowa

**Affiliations:** 1 Department of Analytical Chemistry, Medical University of Lublin, Aleje Raclawickie 1, 20-059 Lublin, Poland; i.sowa@umlub.pl; 2 Department of Plastic Surgery, Specialist Medical Centre, 57-320 Polanica-Zdrój, Poland; piotr_drozdowski@wp.pl; 3 Department of Technology of Cosmetic and Pharmaceutical Products, Medical College, University of Information Technology and Management in Rzeszow, Sucharskiego 2, 35-225 Rzeszow, Poland; aziemlewska@wsiz.edu.pl (A.Z.); mazagorska@wsiz.edu.pl (M.Z.-D.); zniziol@wsiz.edu.pl (Z.N.-Ł.); 4 Department of Biochemistry and General Chemistry, Medical College, University of Rzeszów, 2A Kopisto St., 35-959 Rzeszów, Poland; tkubrak@ur.edu.pl

**Keywords:** isoflavones, antioxidants, skin cells, phytoestrogens, polyphenols, biochanin A, coumestrol

## Abstract

Isoflavones, belonging to polyphenolic compounds, show structural similarity to natural estrogens, and in this context, they have been extensively studied. Some of them are also applied as cosmetic additives; however, little is known regarding their effects on skin cells. In this investigation, common isoflavones, including genistein, daidzein, glycitein, formononetin, and biochanin A, as well as coumestrol, were evaluated for antioxidant activity and their impact on human skin fibroblasts and keratinocytes. Antioxidant effects were assessed using DPPH, ABTS, and FRAP tests, and the ability to scavenge reactive oxygen species (ROS) was tested in cells with H_2_O_2_-provoked oxidative stress. The impact on the activity of antioxidant enzymes (SOD, CAT, GSH) and lipid peroxidation (MDA) was also explored. As shown by Alamar Blue and neutral red uptake assays, the compounds were not toxic within the tested concentration range, and formononetin and coumestrol even demonstrated a stimulatory effect on cells. Coumestrol and biochanin A demonstrated significant antioxidative potential, leading to a significant decrease in ROS in the cells stimulated by H_2_O_2_. Furthermore, they influenced enzyme activity, preventing depletion during induced oxidative stress, and also reduced MDA levels, demonstrating protection against lipid peroxidation. In turn, genistein, daidzein, and glycitein exhibited low antioxidant capacity.

## 1. Introduction

**Figure 1:**
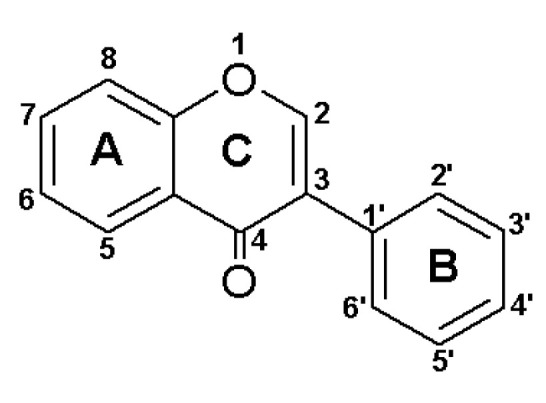
General structure of isoflavones.

Isoflavones are polyphenolic compounds commonly found in various plant species within the *Leguminosae* family. They are distinguished from flavonoids by a distinct arrangement of the molecule, involving the binding of the second benzene ring to the heterocyclic pyran at position 3 (Figure 1), unlike flavonoids, where the binding is at position 2.

They exhibit structural similarity to natural estrogen, 17ß-estradiol. Consequently, they possess mild estrogenic activity and have found application as hormone replacement therapy in postmenopausal women [1,2]. Because of these properties, they are called phytoestrogens. The activity of these compounds has been the subject of extensive research, both in vitro and in vivo, and the health-promoting potential of orally administered isoflavones, particularly those derived from soy, is widely documented in numerous scientific papers [3]. These studies also emphasize the benefits of dietary intake of isoflavones on skin health, e.g., improvement in the thickness of collagen [4,5].

In turn, much less is known about the action of topically applied isoflavones, and only the protective properties against UVB radiation are well evidenced. For example, it has been demonstrated that soy-derived isoflavones, both individually and in a mixture, increased cell viability of UVB-irradiated cells [6], decreased UVB-induced DNA damage, and exhibited an anti-inflammatory effect [7,8,9]. Photoprotective action was also demonstrated in animal models [10,11,12]. Other positive effects on the skin, including increased hyaluronic acid content, improved elasticity, and hydration, have been observed in hairless mice for fermented soy milk extract containing genistein and daidzein [13,14]. In turn, a human study indicated that a cream containing glycine is useful in treating physiological aging and photoaging in postmenopausal women [15]. Moreover, gel with genistein significantly increased the concentration of hyaluronic acid [16], facial skin collagen [17], and epidermal thickness [18].

The antioxidant activity of isoflavones is another important aspect, considering their dermal application. Antioxidants play a crucial role in skin protection against harmful effects of free radicals generated by UV radiation and environmental pollutions which can damage skin cells and accelerate the aging process. They support skin health by preventing oxidative stress, reducing inflammation, and promoting collagen production [19]. There are some reports regarding the antioxidant effects of isoflavones. Generally, tests based on chemical reactions, including DPPH, ABTS, or FRAP reagents, showed only low or moderate activity. Genistein appears to exhibit the greatest potency in scavenging activity of reactive oxygen species (ROS) compared with other soy isoflavones [20,21,22]; however, the data are ambiguous [23].

In the literature, there is a lack of data on the antioxidant potential of phytoestrogens in biological systems, with the exception of Jeon et al., who indicated a potent effect of coumestrol in reducing H_2_O_2_-induced intracellular ROS [23]. Furthermore, the papers evaluating the impact on skin cells are limited to the cytotoxicity of daidzein, genistein, and biochanin A towards human skin fibroblasts [8,24,25].

It should be pointed out that, among phytoestrogens, genistein and daidzein have been the most extensively investigated [16,17,26] and knowledge about the other, structurally similar compounds is scarce. As isoflavones are considered a valuable additive to dermal preparations such as creams or gels [13,15,16], investigating their effects on skin is important from the perspective of safety and understanding possible mechanisms of action that could justify their topical application. Therefore, in this paper, we aimed to evaluate the cytotoxic activity and antioxidant potential of the most common phytoestrogens including genistein, daidzein, glycitein, formononetin, biochanin A, and coumestrol using two types of human skin cells: fibroblasts and keratinocytes. The impact on the activity of antioxidant enzymes (SOD, CAT, GSH) and lipid peroxidation (MDA) was also explored.

## 2. Results and Discussion

**Figure 2:**
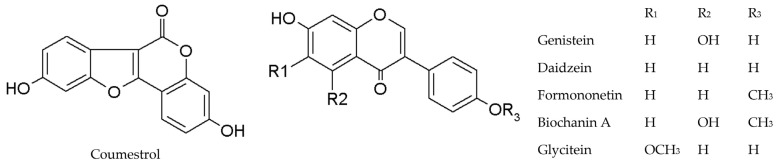
The chemical structures of investigated phytoestrogens.

The investigation involved the aglycone forms of isoflavones found in soy, including genistein, daidzein, glycitein, formononetin, and biochanin A. Coumestrol, belonging to the coumestane class, was also included due to its structural similarity to isoflavones and its phytoestrogenic properties (Figure 2).

### 2.1. Cytotoxicity Assessment

The safety of cosmetic additives is crucial for protecting consumer health, ensuring regulatory compliance, avoiding allergic reactions, and supporting innovation in the cosmetics industry. Cosmetics, as preparations applied directly to skin cells, require assessment for potential toxic effects of their ingredients, especially considering that they can penetrate and impact the deeper layers of the skin. The results of cytotoxicity tests are helpful for the European Commission and its advisory body, the Scientific Committee on Consumer Safety. This committee forms opinions on cosmetic ingredients based on research and scientific publications, allowing the development of guidelines for the introduction of regulations for individual cosmetic ingredients. Thus, assessing the impact of cosmetic ingredients on skin cells is the primary step to consider for their possible application.

**Figure 3:**
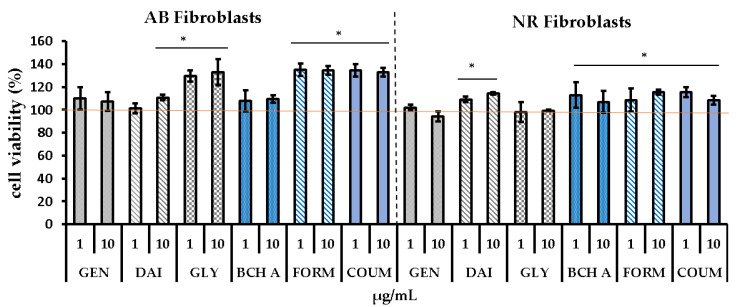

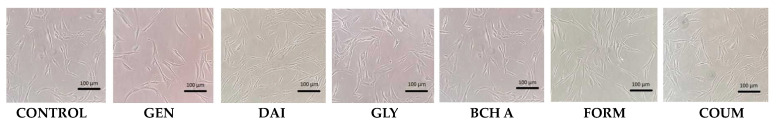
The effects of 24 h exposure to isoflavones (1 and 10 µg/mL) on resazurin reduction (Alamar Blue assay—AB) and neutral red (NR) dye uptake in cultured human skin fibroblasts. Data are mean ± SD; * means the differences were statistically significant (at *p* < 0.05) compared with the control taken as 100%. The images show cultured cells treated with investigated phytoestrogens at concentrations of 10 µg/mL. Images were taken using an inverted microscope at ×10 magnification (scale bar: 100 μm). GEN—genistein, DAI—daidzein, GLY—glycitein, BCH A—biochanin A, FORM—formononetin, COUM—coumestrol.

**Figure 4:**
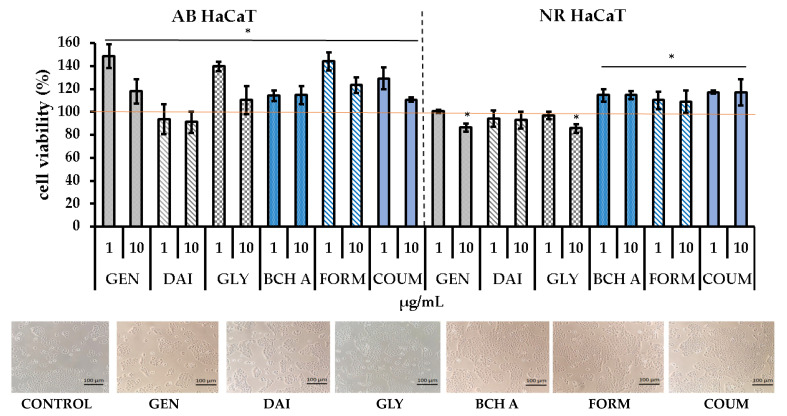
The effects of 24 h exposure to isoflavones (1 and 10 µg/mL) on resazurin reduction (Alamar Blue assay—AB) and neutral red (NR) dye uptake in cultured keratinocytes (HaCaT). Data are the mean ± SD; * means the differences were statistically significant (at *p* < 0.05) compared with the control taken as 100%. The images show cultured cells treated with investigated phytoestrogens at concentrations of 10 µg/mL. GEN—genistein, DAI—daidzein, GLY—glycitein, BCH A—biochanin A, FORM—formononetin, COUM—coumestrol.

In our study’s initial phase, we evaluated the cytotoxic effects of isoflavones using two complementary tests: Alamar Blue (AB) and neutral red (NR) assays. AB is based on the reduction of resazurin to a fluorescent form in response to cellular metabolic processes, while NR utilizes the ability of viable cells to uptake NR dye into lysosomes. We investigated two skin cell lines, including human skin fibroblasts (BJ) and keratinocytes (HaCaT). The results are shown in Figure 3 and Figure 4, respectively.

The isoflavones at the tested concentration range were nontoxic for BJ cells, and only a slight decrease in cell viability was observed in HaCaT for daidzein, based on AB and NR tests, and for genistein and glycitein in the NR assay. In contrast, biochanin A, formononetin, and coumestrol even showed a stimulatory effect on cell viability in both BJ and HaCaT cells, as evidenced by both Alamar Blue and neutral red assays. A slight increase in cell viability was also noted for BJ in the case of daidzein (AB, NR assays) and glycitein (NR assay).

The lack of cytotoxicity of some isoflavones on human skin fibroblast cells at concentrations similar to those used in our study was also noted in the literature [8,24,25]. Iovine et al. found that daidzein and genistein in the range of 10 to 60 μM (2.5–16 μg/mL) did not negatively affect BJ-5ta cells (trypan blue test) [8]. Additionally, Borawska et al. observed that, apart from the lack of cytotoxicity of genistein at a concentration of 1–50 uM (MTT assay), it showed a slight stimulatory effect on normal human skin fibroblasts (NHSFs) at lower concentrations (up to 10 μM), where the cell viability increased by approximately 10% compared with the control [24]. Biochanin A, in the range of 2–100 μM, also showed no cytotoxicity in the MTT test against normal fibroblast cells (NIH3T3) [25].

On the other hand, Pawlicka et al. reported that higher concentrations of genistein (above 50 μM) were detrimental to fibroblasts when exposure time was 48 h [27]. In addition, Chiu et al. found that genistein at 10 µg/mL negatively affects keratinocytes and decreases the cells’ viability to approx. 70% compared with the control [10]. Similarly, we also observed a statistically significant decrease in the viability of keratinocytes in the NR assay.

### 2.2. Assessment of Antioxidant Activity

ROS scavenging activity is a desirable property of cosmetic additives. Free radicals generated under the influence of various environmental factors have a negative impact on the skin’s condition. Excessive ROS production leads to accelerated aging due to unfavorable processes in the skin, including the degradation of collagen fibers, lipid peroxidation, and development of inflammation [28,29]. In a physiological state, antioxidant enzymes such as superoxide dismutase (SOD), catalase (CAT), or glutathione peroxidase (GPx) maintain cellular homeostasis and prevent oxidative stress [30]. However, in the presence of excessive ROS, external antioxidants can support the intracellular system, making them important components in cosmetics [31,32].

#### 2.2.1. DPPH, ABTS, and FRAP Tests

**Figure 5:**
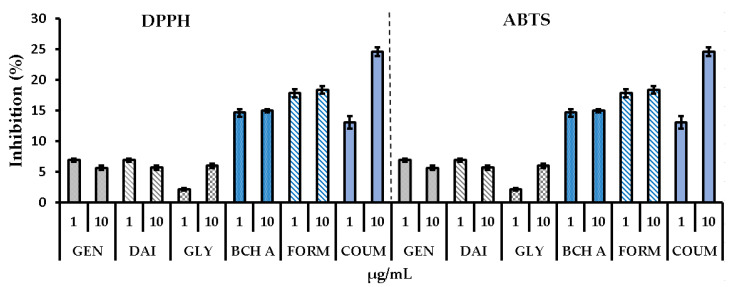
Antioxidant activity of the tested compounds assessed using the DPPH and ABTS assays and expressed as a percentage of inhibition. Data are the mean ± SD. GEN—genistein, DAI—daidzein, GLY—glycitein, BCH A—biochanin A, FORM—formononetin, COUM—coumestrol.

DPPH and ABTS assays were conducted to assess the free radical scavenging capacity of the phytoestrogens. The two tests are considered complementary, providing insights into the potential to neutralize free radicals through electron/hydrogen transfer. The obtained values (Figure 5) demonstrated low antioxidant capacities of genistein, daidzein, and glycitein. The highest activity was noted for coumestrol at a concentration of 10 µg/mL, while formononetin and biochanin A exhibited similar moderate effects.

**Figure 6:**
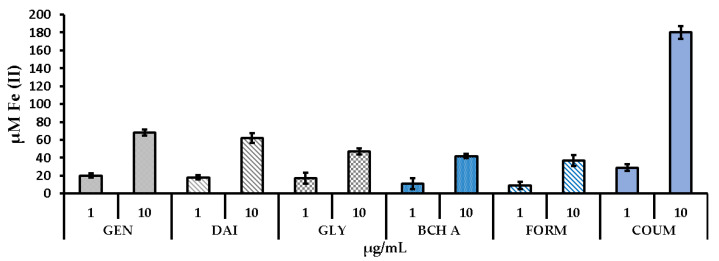
Ferric reducing/antioxidant power (FRAP) of the tested compounds. Data are the mean ± SD. GEN—genistein, DAI—daidzein, GLY—glycitein, BCH A—biochanin A, FORM—formononetin, COUM—coumestrol.

To complete the investigation of the antioxidant potential of the phytoestrogens, a ferric reducing/antioxidant power (FRAP) assay was carried out. This test measures the ability of samples to reduce Fe^3+^ to Fe^2+^. The obtained results (Figure 6) showed a low ferric reducing power of the phytoestrogens, with the exception of coumestrol, which exhibits approximately three times higher activity than the other tested compounds.

#### 2.2.2. Intracellular Reactive Oxygen Species (ROS) Levels

The ability of the tested compounds to eliminate reactive oxygen species (ROS) was assessed in a state of induced oxidative stress, with hydrogen peroxide used as an inducer. The cells were pretreated with phytoestrogens, followed by H_2_O_2_ stimulation.

**Figure 7:**
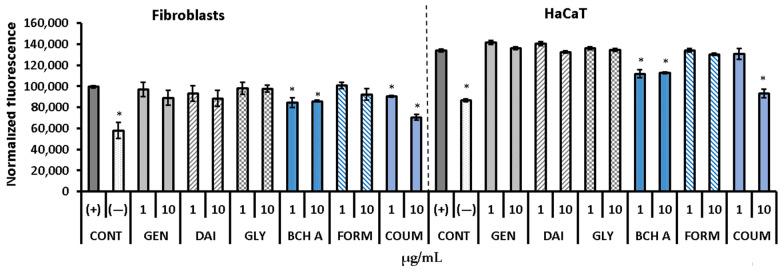
Relative fluorescence of 2′,7′-dichlorodihydrofluorescein (DCF) in human skin fibroblasts and keratinocytes (HaCaT) with induced oxidative stress. The data are means ± SD. * indicates a statistically significant difference vs. H_2_O_2_-stimulated cells (CONT+). One-way ANOVA followed by Dunnett’s post hoc test were used (*p* < 0.05). (CONT−)—untreated cells, GEN—genistein, DAI—daidzein, GLY—glycitein, BCH A—biochanin A, FORM—formononetin, COUM—coumestrol.

In general, the obtained results are in agreement with the DPPH test, as genistein, daidzein, and glycitein did not decrease the ROS level in either tested cell type. Biochanin and coumestrol significantly diminished the increased level of ROS caused by the action of H_2_O_2_, and this effect was observed in both keratinocytes and fibroblasts (Figure 7). In turn, formononetin, which showed free radical scavenging activity in the DPPH test, did not decrease ROS in the H_2_DCFDA assay.

Our study showed that genistein, daidzein, and glycitein had low antioxidant potential in both the DPPH and ABTS tests as well as in cultured human skin fibroblasts and keratinocytes. The weak antioxidant activity of these compounds is also evidenced by recent reports. Arora et al. highlighted the significance of the number and position of hydroxyl groups in antioxidant effectiveness. Genistein, featuring hydroxyl groups at the C-5, C-7, and C-4′ positions, exhibited the highest activity among soy isoflavones, followed by daidzein, with hydroxyl groups at the C-7 and C-4′ positions. Hydroxyl substitution at the C-4′ position was identified as the most crucial, while the C-5 position had a moderate impact, and the C-7 position was deemed less important [21]. The effect was observed in ABTS, ORAC, and DPPH assays [22,33,34]. In addition, these isoflavones have a weak ability to reduce ferric ions (FRAP) [22,34], and the FRAP values at a concentration of 12 µM were below 0.05 mM [34]. In turn, biochanin A and coumestrol showed significant ROS scavenging activity in BJ and HaCaT cells as well as being effective in scavenging of DPPH and ABTS radicals. This is in accordance with the findings of Jeon et al., who demonstrated that coumestrol exhibits significantly higher antioxidant activity than genistein and daidzein. They observed that preincubation of HepG2 cells with coumestrol at a concentration of 1 μM before exposure to H_2_O_2_ decreased the ROS levels from 250% to approx. 150% (non-H_2_O_2_ stimulated cells were considered as 100%) [23]. In our study, only coumestrol showed significant ferric reducing ability, which is in line with the observations of Mitchell et al., who found that the potential of this compound is three or four times higher than that of the other soy isoflavones [22].

#### 2.2.3. Antioxidant Enzyme Activity

**Figure 8:**
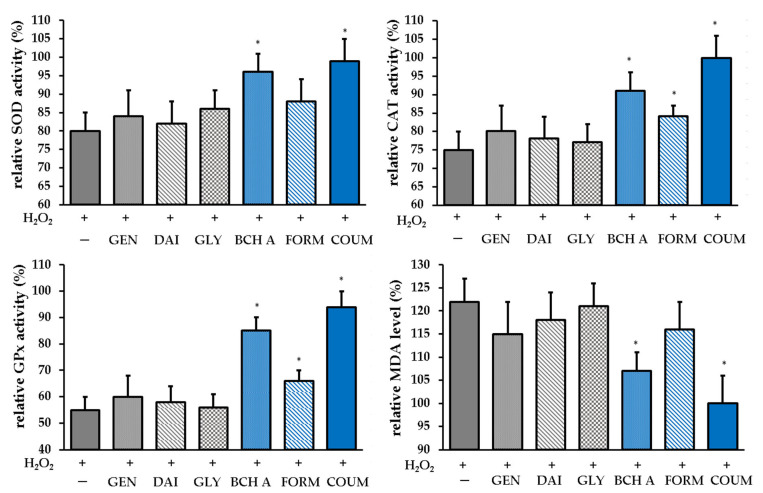
Effect of the phytoestrogen pretreatment prior to the H_2_O_2_ induction on the antioxidant enzyme activity calculated as a percentage of the untreated control (taken as 100%). SOD—superoxide dismutase, CAT—catalase, GPx—glutathione peroxidase, MDA—malondialdehyde. GEN—genistein, DAI—daidzein, GLY—glycitein, BCH A—biochanin A, FORM—formononetin, COUM—coumestrol. The data are means ± SD. * indicates a statistically significant difference (*p* < 0.05) vs. the H_2_O_2_-treated cells. One-way ANOVA followed by Dunnett’s multiple comparison post hoc test were used.

In addition to the direct antioxidant capacity associated with the ability to transfer hydrogen atoms/electrons from the hydroxyl groups to free radicals, some phenolic compounds may affect the activity of antioxidant enzymes. The cellular antioxidant enzyme system is pivotal in an effective defense against oxidative stress, and alterations in the activities of the enzymes such as superoxide dismutase (SOD), catalase (CAT), and glutathione peroxidase (GPx) can serve as indicators of the antioxidant response [35]. Another biomarker of oxidative stress is malondialdehyde (MDA), which is the final product of lipid peroxidation [36]. Therefore, the impact of the tested phytoestrogens on SOD, CAT, and GPx activity and MDA level was investigated. Figure 8 displays the obtained results. As can be seen, only biochanin A and coumestrol significantly affected the enzyme activity and prevented the depletion of enzymes in the state of induced oxidative stress. A slight impact on catalase was also observed for formononetin. Coumestrol and biochanin A also decreased the MDA level.

Our work showed that among the tested phytoestrogens, only biochanin A and coumestrol significantly affect antioxidant enzymes and prevent lipid peroxidation caused by oxidative stress. Jeon et al. also found that pretreatment with coumestrol at a concentration of 0.1 μM decreased MDA content in HepG2 cells, which was elevated by 36.7% after H_2_O_2_ exposition, to control levels, indicating protection against lipid peroxidation. Furthermore, at a concentration of 1 μM, it restored GSH and SOD to control levels, which were depleted under oxidative stress conditions (up to approx. 80% and 40% compared with untreated cells, respectively) [23]. The minor effect of soy isoflavones on the antioxidant enzyme system was also evidenced in an in vivo study using a rat model. Duchnik et al. observed no statistically significant effect on SOD and GPx activity, and only CAT was lower in the skin of rats exposed to the isoflavone mixture [37]. However, it should be mentioned that the literature data are ambiguous. Wang et al. reported that genistein effectively down-regulates the intracellular level of MDA and increases SOD activity under UVB stress conditions in human dermal fibroblasts [38]; however, they used higher concentrations of the compound (up to 80 µg/mL), which were cytotoxic in our experimental conditions.

## 3. Materials and Methods

### 3.1. Materials and Equipment

All isoflavone standards and reagents were purchased from Sigma-Aldrich (St. Louis, MO, USA). Spectrophotometric measurements were conducted using a FilterMax F5 microplate reader spectrophotometer (Thermo Fisher Scientific, Waltham, MA, USA).

### 3.2. Cytotoxicity Analysis

#### 3.2.1. Cell Culture

To assess cytotoxicity, two normal human skin cell lines were used: keratinocytes (HaCaT, CLS Cell Lines Service (Eppelheim, Germany)) and fibroblasts (BJ, ATCC^®^CRL-2522™ American Type Culture Collection, Manassas, WV, USA). DMEM (Dulbecco’s Modified Eagle Medium, Biological Industries, Cromwell, CO, USA) was supplemented with l-glutamine, 4.5 g/L glucose, and sodium pyruvate. The medium was additionally supplemented with 10% (*v*/*v*) fetal bovine serum (FBS, Biological Industries, Beit-Haemek, Israel) and 1% (*v*/*v*) with antibiotics (100 U/mL penicillin and 1000 μg/mL streptomycin, Thermo Fisher Scientific, Waltham, MA, USA). After reaching appropriate confluence, HaCaT and BJ cells were transferred to 96-well plates for 24 h. After this time, the medium was replaced with the tested isoflavones at concentrations of 1 and 10 µg/mL and incubated for 24 h. The plates with the tested compounds were subjected to cytotoxicity tests.

#### 3.2.2. Alamar Blue Assay

The Alamar Blue test was performed according to the procedure described by Page et al. with modifications [39]. After 24 h of incubation of the skin cells with the tested isoflavones, the compounds were aspirated and a resazurin solution (Merck KGaA, Darmstadt, Germany) was added to each well at a concentration of 60 μM. Untreated cells cultured in DMEM were used as controls. The plates were then incubated for 2 h. After this time, fluorescence was measured at λ = 570 nm. Each sample was tested in triplicate.

#### 3.2.3. Neutral Red Uptake Assay

The neutral red uptake test was performed according to the procedure described by Borrenfreund et al. with modifications [40]. After 24 h of incubation, neutral red dye dissolved in DMEM was added to each well of a 96-well plate containing skin cells with the tested isoflavones and incubated for 2 h. Then, the cells were washed with sterile PBS, and 150 μL of destaining buffer (C_2_H_5_OH/CH_3_COOH/H_2_O, 50%/1%/49%) was added. Absorbance was measured at λ = 570 nm. Cells not treated with the tested compounds were used as a control.

### 3.3. Determination of Antioxidant Properties

#### 3.3.1. DPPH and ABTS Scavenging Assays

2,2′-azino-bis(3-ethylbenzothiazoline-6-sulfonic acid) (ABTS•+) and 1,1-diphenyl-2- picrylhydrazyl (DPPH) radicals were used to determine the radical scavenging activity following the methodology described elsewhere [41]. Samples of isoflavones at concentrations of 1 and 10 µg/mL were mixed with methanolic solutions of DPPH or ABTS. Absorbance (Abs.) was measured at λ = 517 nm and λ = 734 nm, respectively. Ascorbic acid was used as a positive control, distilled water as a negative control, and isoflavone samples without DPPH as a blank. The radical scavenging activity was expressed as a percentage of inhibition using the following equation:

$$\%scavenging=\frac{Ac-As}{As}\times 100$$

where As—absorbance of the sample; Ac—absorbance of the control sample.

#### 3.3.2. Ferric Ion Reducing/Antioxidant Power (FRAP) Assay

The ferric reducing abilities of the specimens were evaluated based on the method described in the literature [42]. Each sample was mixed with fresh FRAP reagent, which comprised a mixture of 300 mM acetate buffer at pH 3.6, 10 mM TPTZ dissolved in 40 mM HCl, and 20 mM FeCl_3_·6H_2_O, with proportions set at 10:1:1. The absorbance was then measured at a wavelength of 593 nm.

#### 3.3.3. Detection of Intracellular Levels of Reactive Oxygen Species (ROS)

To determine intracellular ROS levels in HaCaTs and fibroblasts, cells were seeded in 96-well plates at a density of 1 × 10^4^ cells per well and cultured for 24 h. Then, the analyzed isoflavones were placed at concentrations of 1 and 10 µg/mL and incubated for 24 h. After this time, the test samples were replaced with the fluorogenic dye H_2_DCFDA and oxidative stress was induced by adding H_2_O_2_ solution at a final concentration of 500 µM and the samples were incubated for 60 min at 37 °C. H_2_O_2_-treated cells were used as a positive sample. Control samples were cells not treated with the test compounds and H_2_O_2_. Fluorescence was measured at a maximum excitation of 485 nm and emission spectra of 530 nm [43]. Relative activity was expressed in relative fluorescence units (RFUs), and this value was corrected for background fluorescence.

#### 3.3.4. Determination of Antioxidant Enzyme Activity

The methodology involves utilizing a colorimetric kit from Abcam (Berlin, Germany) for the assessment of superoxide dismutase (SOD), catalase (CAT), and glutathione peroxidase (GPx) activity. The procedures were conducted following the manufacturer’s instructions. In the SOD assay, superoxide anions react with tetrazolium salt WST-1 to produce a water-soluble formazan dye, which is detected at 450 nm. In the catalase (CAT) activity assay, the catalase reacts with hydrogen peroxide (H_2_O_2_) to form water and oxygen. The untransformed H_2_O_2_ reacts with the probe and a product can be measured at OD 570 nm. The GPx assay directly measures NADPH consumption in enzyme-coupled reactions. The optical density was measured at 340 nm. In the tests, the decrease in absorbance is directly proportional to the activity of the enzyme in the sample. In the MDA kit, lipid peroxidation is determined by the reaction of malondialdehyde (MDA) with thiobarbituric acid (TBA) to form a colorimetric product detected at 532 nm.

Enzyme activities were calculated according to the formulas available in the protocols.

### 3.4. Statistical Analysis

Each sample was tested in triplicate in three independent experiments. The values measured were expressed as mean ± standard deviation (SD). An analysis of variance (ANOVA) and a post hoc Dunnett’s test were performed. Statistical significance was determined at * *p* < 0.05. Statistical analyses of the obtained results were performed using GraphPad Prism 8.4.3 (GraphPad Software, Inc., San Diego, CA, USA).

## 4. Conclusions

Phytoestrogens are promising cosmetic additives; however, the number of studies regarding their impact on skin cells is limited. Our studies expand the knowledge regarding the cytotoxicity and antioxidant activity of phytoestrogens in biological systems, encompassing keratinocytes and fibroblasts, and provide data on the impact of these compounds on the antioxidant enzyme system. Our investigation revealed that within the tested range, the studied phytoestrogens were nontoxic, and some of them, particularly biochanin A, formononetin, and coumestrol, even demonstrated proliferative activity. However, it was found that genistein, daidzein, and glycitein had low antioxidant potential in both DPPH and ABTS tests as well as in cultured human skin fibroblasts and keratinocytes. Therefore, further investigation into alternative mechanisms of action should be conducted. In turn, biochanin A and coumestrol showed significant ROS scavenging activity in BJ and HaCaT cells as well as being effective in the reduction of DPPH and ABTS. In addition, among the tested phytoestrogens, only biochanin A and coumestrol significantly affect antioxidant enzymes and prevent lipid peroxidation caused by oxidative stress. Thus, these phytoestrogens may be considered as valuable antioxidants for potential cosmetic application.

## Data Availability

The data presented in this study are available on request from the corresponding author.

## References

[1] Aboushanab S.A., Khedr S.M., Gette I.F., Danilova I.G., Kolberg N.A., Ravishankar G.A., Ambati R.R., Kovaleva E.G. (2023). Isoflavones Derived from Plant Raw Materials: Bioavailability, Anti-Cancer, Anti-Aging Potentials, and Microbiome Modulation. Crit. Rev. Food Sci. Nutr..

[2] Vitale D.C., Piazza C., Melilli B., Drago F., Salomone S. (2013). Isoflavones: Estrogenic Activity, Biological Effect and Bioavailability. Eur. J. Drug Metab. Pharmacokinet..

[3] Kim I.-S. (2021). Current Perspectives on the Beneficial Effects of Soybean Isoflavones and Their Metabolites for Humans. Antioxidants.

[4] Liu T., Li N., Yan Y., Liu Y., Xiong K., Liu Y., Xia Q., Zhang H., Liu Z. (2020). Recent Advances in the Anti-aging Effects of Phytoestrogens on Collagen, Water Content, and Oxidative Stress. Phytother. Res..

[5] Polito F., Marini H., Bitto A., Irrera N., Vaccaro M., Adamo E.B., Micali A., Squadrito F., Minutoli L., Altavilla D. (2012). Genistein Aglycone, a Soy-derived Isoflavone, Improves Skin Changes Induced by Ovariectomy in Rats. Br. J. Pharmacol..

[6] Huang Z.-R., Hung C.-F., Lin Y.-K., Fang J.-Y. (2008). In Vitro and in Vivo Evaluation of Topical Delivery and Potential Dermal Use of Soy Isoflavones Genistein and Daidzein. Int. J. Pharm..

[7] Moore J.O., Wang Y., Stebbins W.G., Gao D., Zhou X., Phelps R., Lebwohl M., Wei H. (2006). Photoprotective Effect of Isoflavone Genistein on Ultraviolet B-Induced Pyrimidine Dimer Formation and PCNA Expression in Human Reconstituted Skin and Its Implications in Dermatology and Prevention of Cutaneous Carcinogenesis. Carcinogenesis.

[8] Iovine B., Iannella M.L., Gasparri F., Monfrecola G., Bevilacqua M.A. (2011). Synergic Effect of Genistein and Daidzein on UVB-Induced DNA Damage: An Effective Photoprotective Combination. J. Biomed. Biotechnol..

[9] Iovine B., Iannella M.L., Gasparri F., Giannini V., Monfrecola G., Bevilacqua M.A. (2012). A Comparative Analysis of the Photo-Protective Effects of Soy Isoflavones in Their Aglycone and Glucoside Forms. Int. J. Mol. Sci..

[10] Chiu T.-M., Huang C.-C., Lin T.-J., Fang J.-Y., Wu N.-L., Hung C.-F. (2009). In Vitro and in Vivo Anti-Photoaging Effects of an Isoflavone Extract from Soybean Cake. J. Ethnopharmacol..

[11] Brand R.M., Jendrzejewski J.L. (2008). Topical Treatment with (−)-Epigallocatechin-3-Gallate and Genistein after a Single UV Exposure Can Reduce Skin Damage. J. Dermatol. Sci..

[12] Shyong E.Q., Lu Y., Lazinsky A., Saladi R.N., Phelps R.G., Austin L.M., Lebwohl M., Wei H. (2002). Effects of the Isoflavone 4′,5,7-Trihydroxyisoflavone (Genistein) on Psoralen plus Ultraviolet A Radiation (PUVA)-Induced Photodamage. Carcinogenesis.

[13] Miyazaki K., Hanamizu T., Sone T., Chiba K., Kinoshita T., Yoshikawa S. (2004). Topical Application of Bifidobacterium-Fermented Soy Milk Extract Containing Genistein and Daidzein Improves Rheological and Physiological Properties of Skin. J. Cosmet. Sci..

[14] Miyazaki K., Hanamizu T., Iizuka R., Chiba K. (2003). *Bifidobacterium*-Fermented Soy Milk Extract Stimulates Hyaluronic Acid Production in Human Skin Cells and Hairless Mouse Skin. Skin Pharmacol. Physiol..

[15] Giorgini S., Greco A., Cristina Melli M., Lotti T.M. (2006). Phytoestrogens in Dermatocosmetology. Clinical and Instrumental Trial Conducted on a Cream Made from Glicine and Other Bioflavonoids in Spontaneous Menopausal Women. Ital J Dermatol Venerol..

[16] Patriarca M.T., Barbosa De Moraes A.R., Nader H.B., Petri V., Martins J.R.M., Gomes R.C.T., Soares J.M. (2013). Hyaluronic Acid Concentration in Postmenopausal Facial Skin after Topical Estradiol and Genistein Treatment: A Double-Blind, Randomized Clinical Trial of Efficacy. Menopause.

[17] Silva L.A., Ferraz Carbonel A.A., De Moraes A.R.B., Simões R.S., Sasso G.R.D.S., Goes L., Nunes W., Simões M.J., Patriarca M.T. (2017). Collagen Concentration on the Facial Skin of Postmenopausal Women after Topical Treatment with Estradiol and Genistein: A Randomized Double-Blind Controlled Trial. Gynecol. Endocrinol..

[18] Moraes A.B., Haidar M.A., Soares J.M., Simões M.J., Baracat E.C., Patriarca M.T. (2009). The Effects of Topical Isoflavones on Postmenopausal Skin: Double-Blind and Randomized Clinical Trial of Efficacy. Eur. J. Obstet. Gynecol. Reprod. Biol..

[19] Hoang H.T., Moon J.-Y., Lee Y.-C. (2021). Natural Antioxidants from Plant Extracts in Skincare Cosmetics: Recent Applications, Challenges and Perspectives. Cosmetics.

[20] Ruiz-Larrea M.B., Mohan A.R., Paganga G., Miller N.J., Bolwell G.P., Rice-Evans C.A. (1997). Antioxidant Activity of Phytoestrogenic Isoflavones. Free Radic. Res..

[21] Arora A., Nair M.G., Strasburg G.M. (1998). Antioxidant Activities of Isoflavones and Their Biological Metabolites in a Liposomal System. Arch. Biochem. Biophys..

[22] Mitchell J.H., Gardner P.T., McPhail D.B., Morrice P.C., Collins A.R., Duthie G.G. (1998). Antioxidant Efficacy of Phytoestrogens in Chemical and Biological Model Systems. Arch. Biochem. Biophys..

[23] Jeon H.Y., Seo D.B., Shin H.-J., Lee S.-J. (2012). Effect of *Aspergillus Oryzae*-Challenged Germination on Soybean Isoflavone Content and Antioxidant Activity. J. Agric. Food Chem..

[24] Borawska M.H., Czechowska S.K., Markiewicz R., Hayirli A., Olszewska E., Sahin K. (2009). Cell Viability of Normal Human Skin Fibroblast and Fibroblasts Derived from Granulation Tissue: Effects of Nutraceuticals. J. Med. Food.

[25] Sehdev V., Lai J.C.K., Bhushan A. (2009). Biochanin A Modulates Cell Viability, Invasion, and Growth Promoting Signaling Pathways in HER-2-Positive Breast Cancer Cells. J. Oncol..

[26] Wei H., Saladi R., Lu Y., Wang Y., Palep S.R., Moore J., Phelps R., Shyong E., Lebwohl M.G. (2003). Isoflavone Genistein: Photoprotection and Clinical Implications in Dermatology. J. Nutr..

[27] Pawlicka M.A., Zmorzyński S., Popek-Marciniec S., Filip A.A. (2022). The Effects of Genistein at Different Concentrations on MCF-7 Breast Cancer Cells and BJ Dermal Fibroblasts. Int. J. Mol. Sci..

[28] Pająk J., Nowicka D., Szepietowski J.C. (2023). Inflammaging and Immunosenescence as Part of Skin Aging—A Narrative Review. Int. J. Mol. Sci..

[29] Zuo L., Prather E.R., Stetskiv M., Garrison D.E., Meade J.R., Peace T.I., Zhou T. (2019). Inflammaging and Oxidative Stress in Human Diseases: From Molecular Mechanisms to Novel Treatments. Int. J. Mol. Sci..

[30] Huchzermeyer B., Menghani E., Khardia P., Shilu A. (2022). Metabolic Pathway of Natural Antioxidants, Antioxidant Enzymes and ROS Providence. Antioxidants.

[31] Michalak M. (2022). Plant-Derived Antioxidants: Significance in Skin Health and the Ageing Process. Int. J. Mol. Sci..

[32] Merecz-Sadowska A., Sitarek P., Kucharska E., Kowalczyk T., Zajdel K., Cegliński T., Zajdel R. (2021). Antioxidant Properties of Plant-Derived Phenolic Compounds and Their Effect on Skin Fibroblast Cells. Antioxidants.

[33] Rüfer C.E., Kulling S.E. (2006). Antioxidant Activity of Isoflavones and Their Major Metabolites Using Different in Vitro Assays. J. Agric. Food Chem..

[34] Lee C. (2005). Relative Antioxidant Activity of Soybean Isoflavones and Their Glycosides. Food Chem..

[35] Nandi A., Yan L.-J., Jana C.K., Das N. (2019). Role of Catalase in Oxidative Stress- and Age-Associated Degenerative Diseases. Oxid. Med. Cell. Longev..

[36] Barrera G., Pizzimenti S., Daga M., Dianzani C., Arcaro A., Cetrangolo G.P., Giordano G., Cucci M.A., Graf M., Gentile F. (2018). Lipid Peroxidation-Derived Aldehydes, 4-Hydroxynonenal and Malondialdehyde in Aging-Related Disorders. Antioxidants.

[37] Duchnik E., Kruk J., Baranowska-Bosiacka I., Pilutin A., Maleszka R., Marchlewicz M. (2019). Effects of the Soy Isoflavones, Genistein and Daidzein, on Male Rats’ Skin. Adv. Dermatol. Allergol..

[38] Wang Y.N., Wu W., Chen H.C., Fang H. (2010). Genistein Protects against UVB-Induced Senescence-like Characteristics in Human Dermal Fibroblast by p66Shc down-Regulation. J. Dermatol. Sci..

[39] Page B., Page M., Noel C. (1993). A New Fluorometric Assay for Cytotoxicity Measurements In-Vitro. Int. J. Oncol..

[40] Borenfreund E., Puerner J.A. (1985). Toxicity Determined in Vitro by Morphological Alterations and Neutral Red Absorption. Toxicol. Lett..

[41] Zagórska-Dziok M., Ziemlewska A., Mokrzyńska A., Nizioł-Łukaszewska Z., Sowa I., Szczepanek D., Wójciak M. (2023). Comparative Study of Cytotoxicity and Antioxidant, Anti-Aging and Antibacterial Properties of Unfermented and Fermented Extract of *Cornus Mas* L.. Int. J. Mol. Sci..

[42] Sowa I., Paduch R., Mołdoch J., Szczepanek D., Szkutnik J., Sowa P., Tyszczuk-Rotko K., Blicharski T., Wójciak M. (2023). Antioxidant and Cytotoxic Potential of Carlina Vulgaris Extract and Bioactivity-Guided Isolation of Cytotoxic Components. Antioxidants.

[43] Evangelista-Vargas S., Santiani A. (2017). Detection of Intracellular Reactive Oxygen Species (Superoxide Anion and Hydrogen Peroxide) and Lipid Peroxidation during Cryopreservation of Alpaca Spermatozoa. Reprod. Domest. Anim..

